# Important topics in the future of biomaterials and stem cells for bone tissue engineering: Comments from the participants of the International Symposium on Recent Trend of Biomaterials and Stem Cells for Bone Tissue Engineering at Changchun, China

**DOI:** 10.1093/rb/rbv004

**Published:** 2015-05-25

**Authors:** Peibiao Zhang, QingSan Zhu

**Affiliations:** ^1^Key Laboratory of Polymer Ecomaterials, Changchun Institute of Applied Chemistry, University of Chinese Academy of Sciences, Chinese Academy of Sciences, Changchun, 130022, P.R. China and ^2^Department of Orthopaedics, China-Japan Union Hospital, Jilin University, Changchun, 130033, P.R. China

Fundamental and clinical experts from Japan, Korea and China, warmly gathered on the beautiful ice and snow city—Changchun, the capital of Jilin Province, China—during 23-26 January 2015, to present their research findings and participated in discussion relating to progress in biomaterials, stem cells and bone tissue engineering. The International Symposium on Recent Trend of Biomaterials and Stem Cells for Bone Tissue Engineering (BTE 2015) was hosted by the Key Laboratory of Polymer Ecomaterials, Changchun Institute of Applied Chemistry, Chinese Academy of Sciences, and co-organized by the Department of Orthopaedics, China-Japan Union Hospital, Jilin University. It provided a new platform of academic and technological communication for fundamental researchers and orthopedic surgeons to express their diverse ideas and inspiring new cooperation.

The field of tissue engineering has emerged as an important approach to the clinical therapy of damaged bone due to trauma, tumor resections or congenital anomalies [1–3]. Besides bone forming cells and growth factors, we have profoundly recognized that the development of bone tissue engineering is directly related to changes in materials technology [4, 5]. However, after experiencing the rapid development of bone tissue engineering over the past several decades, we still currently face a tremendous challenge to design a new platform that can integrate bioscaffolds and growth factors or stem cells into biomimetic bone substitutes with new technologies for reconstruction of large-size orthopedic defects [6, 7].

The participants focused on discussing the recent development of synthetic or natural bioscaffolds that can provide 3D architecture [8, 9], appropriate topography and suitable surface chemistry [7, 10, 11], as well as enough mechanical strength [12, 13], which can encourage desired cellular activities and guide bone tissue regeneration. The introduction of bioactive molecules into 3D porous scaffolds physically or chemically to mimic the *in vivo* microenvironment is a promising strategy for tissue engineering and stem cell research [14]. A series of engineered binding growth factors [14–16] or peptides [17] have been designed and employed to study the biomaterial-stem cell interactions and to direct stem cell behaviors. Genetic tissue engineering was developed by bone marrow-derived mesenchymal stem cells (MSCs) reinforced with gene-transfecting for local delivery of bone morphogenetic proteins (BMPs) [18, 19]. The post-materials technology, or named offline materials technology, is also proposed in the symposium for researchers and surgeons to focus on the research of combination of current material products and clinical available resources, which will broaden and accelerate the clinical application of current material products.

We made a concise report on this symposium, and some comments of the key speakers in the symposium were presented. We hope that their comments can arouse further interests of readers in discussing future strategies of bone tissue engineering and regenerative medicine. Moreover, we believed that the frequent appointment of researchers and orthopedic surgeons may inspire more and more ideas in the development of future clinical products of tissue engineering for orthopedic application.

## Yoshihiro Ito


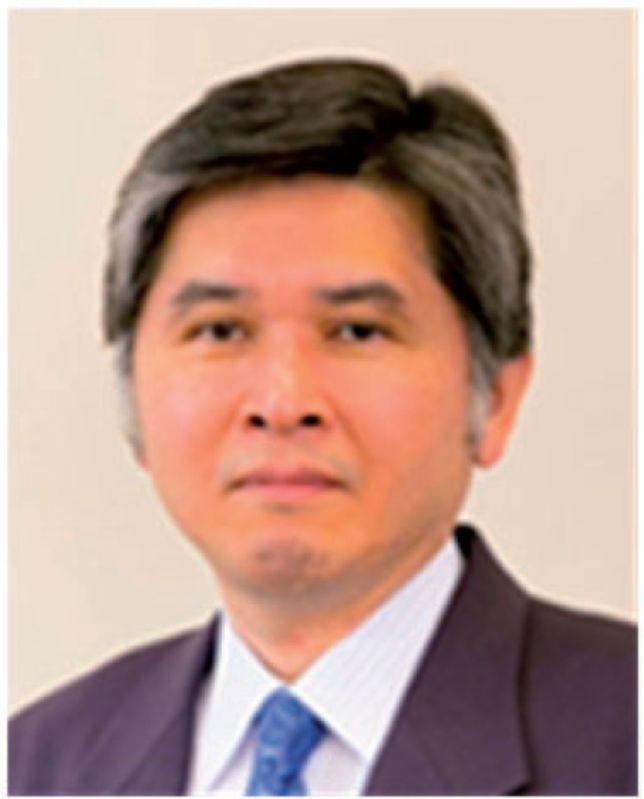


Chief Scientist and Director of the Nano Medical Engineering Laboratory at the RIKEN (from 2004). He received his Bachelor’s degree in polymer chemistry at Kyoto University and was awarded a doctorate in engineering from the same university in 1987. Since then he has held some posts as assistant and associate professor at Kyoto University, research fellow at the University of California, Irvine (1992-93), professor of the University of Tokushima, and Project Leader at the Kanagawa Academy of Science and Technology. Currently, he is also a visiting professor of some universities including Tokyo Institute of Technology, Tokyo Metropolitan University, Hokkaido University and Waseda University. He received the Award of Japanese Society of Biomaterials (2009) and Fellow, Biomaterial Science and Engineering (2012). His research focuses on biomaterial science, regenerative medical engineering, combinatorial bioengineering for the creation of functional polymers and soft nanotechnology.

***Comment:**** The surface properties are of prime importance in establishing the response to tissue from biomaterials. I have investigated the immobilization of growth factor proteins to provide a set of very powerful signals for cells. Immobilized growth factors stimulated cultured cells for a longer time and enhanced cell growth more than did soluble growth factors and also induced cellular differentiation. The effects of immobilized growth factors were visualized by their micropatterning on substrates. Recently, we extended the immobilization technology from chemical to biological one, which is protein engineering for synthesis of binding growth factors. We synthesized some types of growth factors carrying collagen binding sequence extracted from fibronectin. One of them was collagen binding-BMP, which was synthesized by silk worm, and directly injected or utilized with scaffold or visible-light-curable gelatin gel. We demonstrated that both of the direct injection and the bound implants significantly enhanced bone formation in animals. Another type of binding growth factors was also designed by combination with binding motif of underwater adhesive proteins composed of non-canonical amino acids such as phosphorylated serine from salivary statherin and 3,4-dihydroxyphenylalanine from mussel protein for binding to inorganic materials such as metals and ceramics. The technology uses not only usual protein engineering but also bioorthogonal approach. As a result osteogenetic differentiation was enhanced on the bound apatite. These new types of binding growth factor could contribute current treatment methods of various debilitating injuries and diseases and deliver on the therapeutic promise of bone tissue engineering.*

## Inn-Kyu Kang


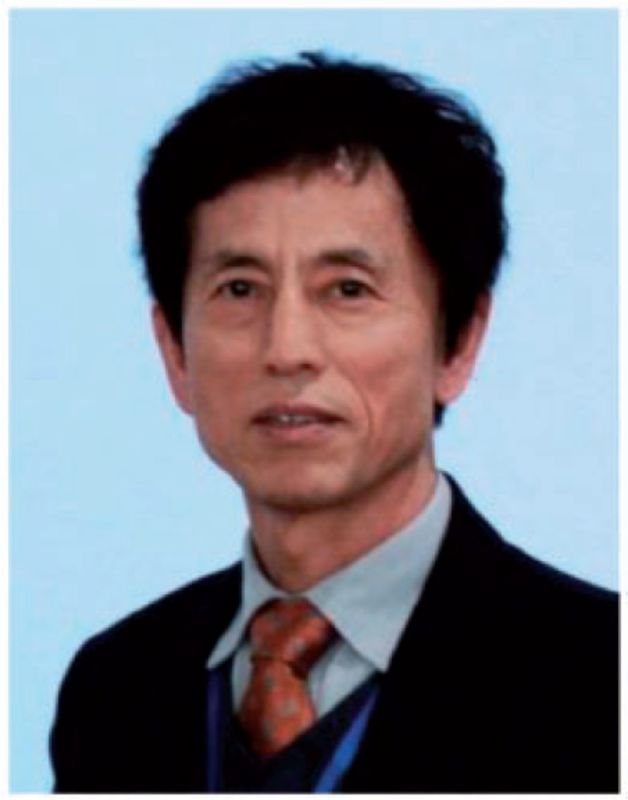


Professor in the Department of Polymer Science and Engineering at the School of Applied Chemical Engineering, Graduate School, Kyungpook National University. He is currently a director of Center for Intelligent Hybrid Nanomaterials and Processes supported by Brain Korea 21+ National Program. He obtained his Ph.D. degree in Polymer Chemistry at Kyoto University in 1987, and joined in the Department of Polymer Science and Engineering in 1988 as a professor. He has published more than 190 articles and his research is focused on surface modification, nanofiber scaffolds, functionalization of nanoparticles and liquid crystal sensors.

***Comment:**** During last one decade several advancements have been made in fabrication of scaffolds for tissue engineering using various techniques of fabrication and materials of different origin. The scaffolds for tissue engineering are ranging from nanofibers to nanoparticles and have been developed by tuning their morphology and architecture. Several breakthroughs have been reported and successful attempts have been made to overcome the drawbacks of the scaffolds fabricated by conventional and modern methods of scaffolds formation yet significant amount of research is required to control the vascularity of the scaffolds, which is a major challenge in the field of fabrication of scaffolds for bone tissue engineering. Although few attempts have been made by harvesting cells from donor/patient to vascularize the scaffolds before their implantation in the patient but area still need intensive research to develop scaffolds with proper vascularization. The worldwide research has achieved success in tissue engineering but matrix induced autologus condrocyte implantation has received little success in repair of cartilage. The bioactivity of scaffolds totally depends on surface topology, microstructure, chemistry and mechanical properties of scaffolds. Thus, controlling the cells behavior and remodeling is a critical step in development of next generation scaffolds for tissue engineering. The functionalized scaffolds loaded with certain amount of growth factor and therapeutics to stimulate cells proliferation and treatment of infection after surgical operations also strongly needed to achieve success in the area of tissue engineering and regeneration. The increasing interest to incorporate a drug delivery function in the scaffolds is emerged as a new field of scaffolds engineering. The drug loaded scaffolds are able to provide sufficient amount of drug at a specific place in comparison to systematic drug delivery systems. The scaffolds loaded with growth factors such as cytokines, hormones, morphogens and proteins need to be developed to enhance cells adhesion, proliferation and differentiation in tissue engineering. The mechanical behavior of scaffolds has important implication on tissue regeneration; stiff scaffolds mimic precalcified bone and elastic scaffolds mimic the elasticity of muscles, which cause MSC differentiation down to a myogenic pathway. Thus, scaffolds with optimized mechanical strength need to be fabricated for enhanced bone tissue engineering. The critical review of the literature reported during last one decade has clearly indicated that there is lot of scope to improve the properties and functionality of the scaffolds for bone tissue engineering using various biomaterials and their fabrication techniques.*

## Guoping Chen


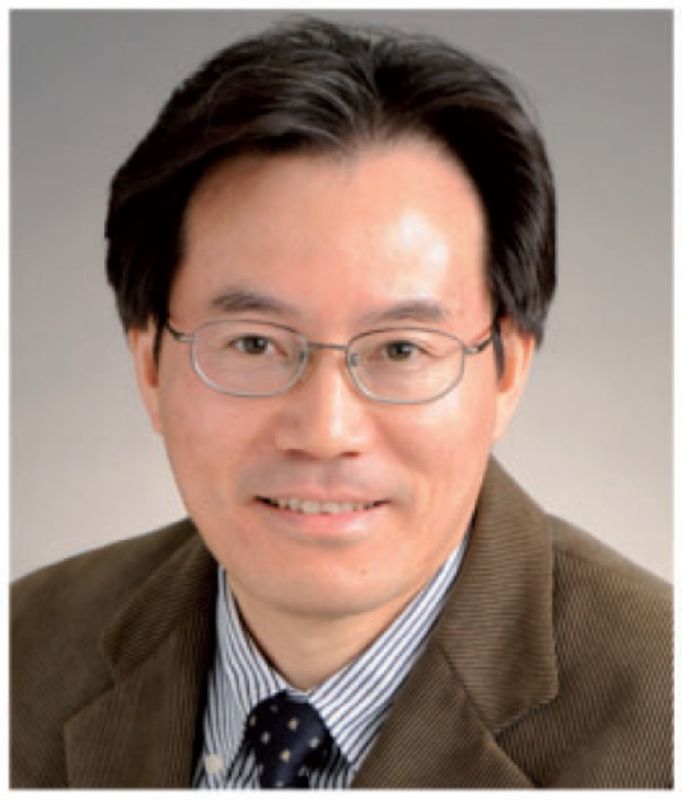


Principal Investigator and Unit Director of Tissue Regeneration Materials Unit, International Center for Materials Nanoarchitectonics, National Institute for Materials Science, Japan. He obtained his Ph.D. from Kyoto University in 1997 majoring in biomaterials and did postdoctoral research until 2000. He became researcher in 2000 and senior researcher in 2003 at Tissue Engineering Research Center, National Institute for Advanced Industrial Science and Technology, Japan. He moved to Biomaterials Center, National Institute for Materials Science as senior researcher in 2004 and was group leader from January, 2007 to March, 2011. He is concurrently Professor of Joint Doctoral Program in Materials Science and Engineering, Graduate School of Pure and Applied Science, University of Tsukuba, Japan. He is Guest Professor of Shanghai Institute of Ceramics, Chinese Academy of Sciences; Southeast University and Sichuan University, China. He is Associate Editor of Journal of Materials Chemistry B; Editor of Science China Chemistry and Editorial Board of Journal of Bioactive and Compatible Polymers and Tissue Engineering (Parts A, B and C).

***Comment:**** Tissue engineering has attracted tremendous endeavors from various fields of material science, biomedical science, chemical engineering, stem cell biology, clinics, industry and etc. The disciplinary research to regenerate functional tissues and organs has reached enormous progress and achievements spanning form fundamental research to clinical applications and industrialization. As one of the tissue engineering, triad, scaffolds not only accommodate implanted cells but also control cell functions to guide functional tissue engineering. Design and creation of highly functional scaffolds have been challenged to mimic the in vivo dynamically remodeling cellular microenvironments. Scaffolds with controlled chemical compositions, nano- and micro-structures, mechanical property and some special functions have been developed form synthetic and naturally derived biomaterials and decellularized matrices. The scaffolds can provide biological and physiochemical cues to recruit stem cells, induce stem cells differentiation and promote regeneration of various tissues and organs. Development of new and highly functional scaffolds will further facilitate efficient regeneration of functional tissues and organs for clinical applications and industrialization.*

## Byung-Soo Kim


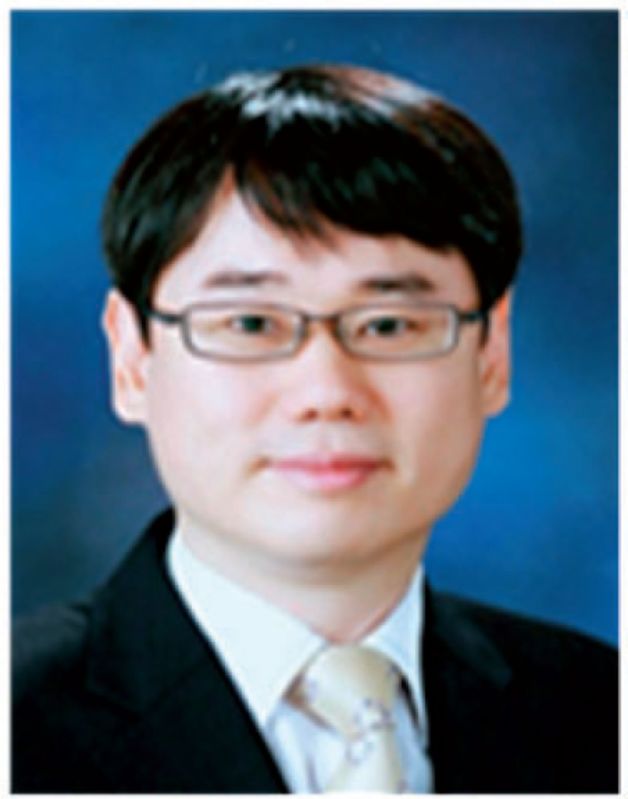


Professor of Chemical and Biological Engineering and a Chairman of Interdisciplinary Program of Bioengineering in Seoul National University, South Korea. He is an active researcher with 275 peer-reviewed publications, 14 book chapters and 17 patents. He has received William B. Walsh Award and Shinyang Award. He had served or now serves as an Associate Editor or an editorial board member of 10 journals including the Tissue Engineering, Journal of Tissue Engineering and Regenerative Medicine, Biotechnology and Bioprocess Engineering, Journal of Industrial and Engineering Chemistry and Tissue Engineering and Regenerative Medicine. He is the chair of Scientific Committee of Korean Tissue Engineering and Regenerative Medicine Society and an organizer of 15th International Biotechnology Symposium.

***Comment:**** Currently, a number of bone graft substitutes have been developed and are being utilized clinically, but there are rooms for improvement. Bone graft substitutes can promote bone regeneration by the interaction of three components: (i) bone-forming cells, (ii) a suitable biomaterial scaffold and (iii) biological stimulants. In the International Symposium on Recent Trend of Biomaterials and Stem Cells for Bone Tissue Engineering (BTE 2015), which was held in Changchun in China in January, 2015, new technologies on these three components for bone regeneration were presented. For large bone-defect, stem cells or progenitor cells need to be implanted since recruitment of bone-forming cells to the large bone-defect may be limited. For effective bone regeneration, implantation of osteogenically induced stem cells or progenitor cells results in better bone regeneration rather than implantation of undifferentiated cells. Thus, decent technologies for ex vivo osteogenic induction of stem cells would be necessary. Meanwhile, biomaterials can be designed in nano scale to present biological stimulants for effective bone regeneration. BMPs are currently used for bone regeneration in patients, but often cause adverse effects due to the high dosage. Drug delivery carrier determines the bone regeneration efficacy of BMPs. Efforts should be made to develop an appropriate delivery carrier which can deliver BMPs without the adverse effects.*

## Xuesi Chen


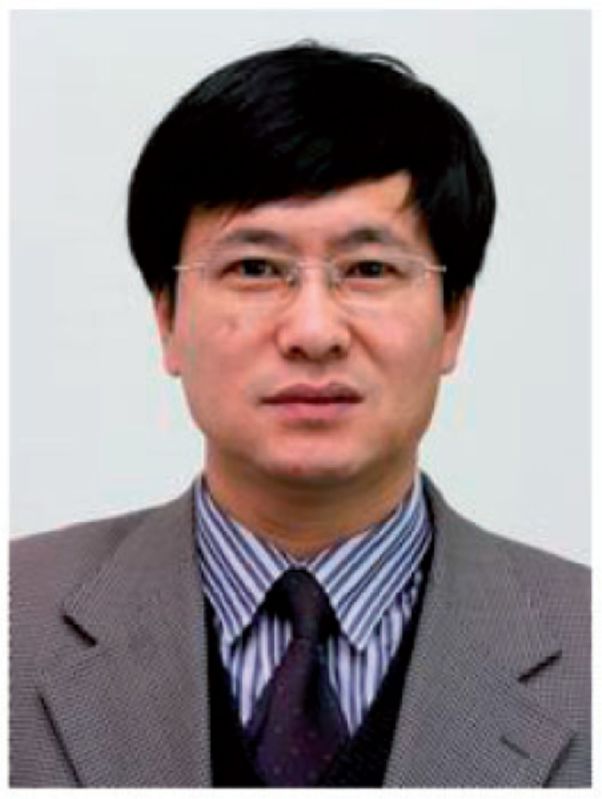


Professor of polymer chemistry in Changchun Institute of Applied Chemistry (CIAC), Chinese Academy of Sciences (CAS) from June 1999. He graduated from the Chemistry Department of Jilin University. He got his Master degree from CIAC in 1988. He got his Ph.D. from Waseda University Japan on March 1997. He had postdoc experience in the University of Pennsylvania from 1997 to 1999. Now he is working in Key Laboratory of Polymer Ecomaterials of CAS in CIAC. He is one of the board members of Journal of Controlled Release, Biomacromolecules, Advanced Healthcare Materials, Acta Biomaterialia and Macromolecular Bioscience. He has published 490 papers.

***Comment:**** In the past 20 years, the regenerative medicine materials, especially the tissue engineering repair materials have made important progress. Tissue engineering is an interdisciplinary field, including materials chemistry and physics, biological engineering, biochemistry, biomechanics, cell biology, biotechnology, clinical medicine, etc. It is one of the important means to repair and rebuild the body’s tissues and organs and has been becoming more and more important. The novel therapy method will change the quality and the way of people’s life, and extend the life of human beings in the near future. Biodegradable polymer material (absorbed in vivo) is one of the very important elements in the process of tissue engineering therapy. As a result, the preparation of porous bioscaffolds with adjustable absorption time, good strength, pore structure and distribution under well control, and good biocompatibility, are the objective requirements for the future development of tissue engineering therapy. We hope that through the joint efforts of scientists, more and more products of tissue engineering devices will be widely used in clinic in the near future.*

## Hongwei Ouyang


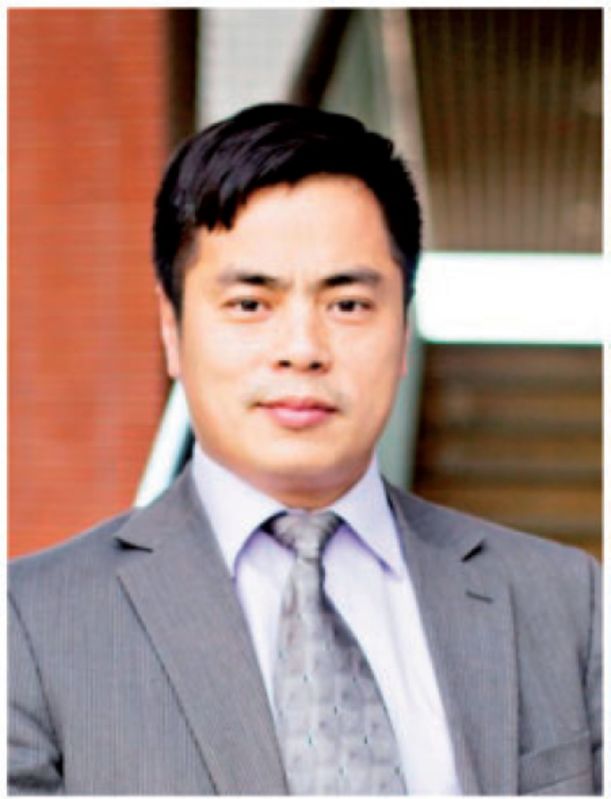


The ‘Qiu-shi’ Distinguished Professor and dean for School of Basic Medical Science, Vice Dean College of Medicine in Zhejiang University, China. He received his Ph.D. degree in orthopedics from National University of Singapore in 2003. Since returned to China in 2005, Prof. Ouyang focuses on the research of tissue engineering and regenerative therapy of musculoskeletal system. His research has been granted support by National Natural Science Foundation of China (NSFC) for Distinguished Young Scholars, The State Key Program of National Natural Science Foundation of China, The National Key Basic Research Program, The National High Technology Research and Development Program of China (863 Program), etc. Currently, he has filed 10 national patents applications (six approved) and published more than 60 original research papers in the international peer review journals. More than 40 papers corresponded by Prof. Ouyang were published in the leading journals of regenerative medicine fields, such as Stem Cells, Advanced Functional Materials/Biomaterials, Annals of the Rheumatic Disease, Tissue Eng, Cell Transplantation. Moreover, he was authorized by State Food and Drug Administration (SFDA) and Ministry Of Health (MOH) to formulate the guidance for assessment of implants for cartilage repair and therapeutic transplantation of engineered tissues, respectively. Prof. Ouyang has established a standard approach for tissue engineered cartilage (TEC) transplantation and is the pioneer of clinical translation in China orthopedic regenerative medicine. Because of his outstanding achievements, Prof. Ouyang has gained great national and international reputation, he is currently the chair of China Orthopedic Regenerative Medicine and the vice chair of China Tissue Engineering and Regenerative Medicine Society. He is also the council member of several international academic societies such as Tissue Engineering and Regenerative Medicine International Society-Asia Pacific Meeting (TERMIS-AP).

***Comment:**** Decades of experience indicated that manufacture of mature tissues with tissue engineering approaches for transplantation is difficult to be achieved. The most feasible and effective way is to apply one or all of the appropriate cell source, biomaterials/scaffold, growth factors according to particular tissues and diseases at the early or middle stage of diseases, which can prevent the tissue/organ from failure and avoid organ transplantation.*

## Qigang Wang


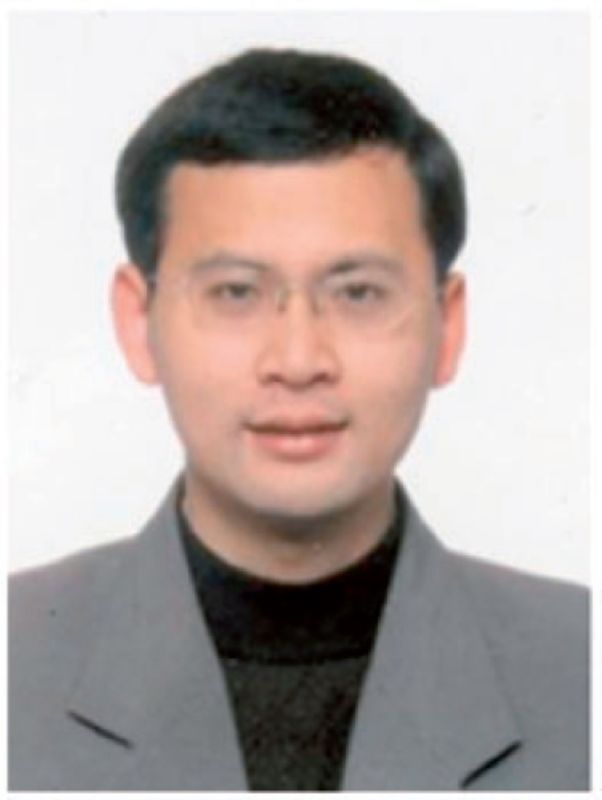


Professor of Department of Chemistry, Tongji University, China. He obtained Ph.D. at the Shanghai Institute of Ceramics (CAS) in 2005, after which he worked as a postdoc in Hong Kong University of Science and Technology, University of Tokyo, and RIKEN. In 2011, he was appointed as a professor at Tongji University. His research interests focus on the mild polymerized preparation of tough nanocomposite gel and the potential applications in tissue engineering and bio-imaging. His current group has published more than 20 Science Citation Index papers, including Advance Materials and Chemical Science.

***Comment:**** Hydrogels with synthetic or natural polymer are useful in cartilage tissue engineering due to their water-swollen networks and suitable microenvironment for tissue formation. Up to now, engineering cartilage tissue still remains a significant challenge due to the low mechanical strength and unsuitable component design. The in-situ hydrogelations via the mild enzyme-mediated radical polymerization can provide a tough artificial cartilage by the direct injection in the defect position.*

## Chengtie Wu


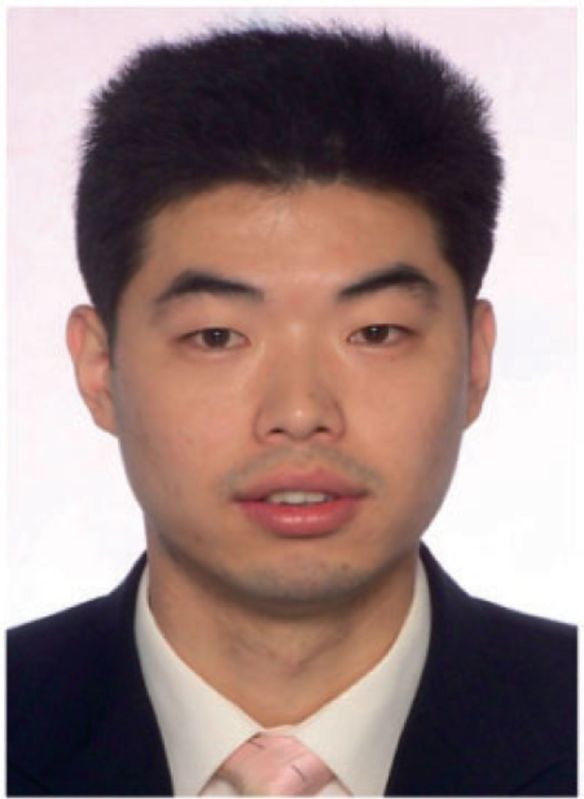


Professor in Shanghai Institute of Ceramics, Chinese Academy of Sciences (SIC, CAS). He completed his Ph.D. in 2006, and then he worked in the University of Sydney, Dresden University of Technology, Germany and Queensland University of Technology where he was awarded Vice-Chancellor Research Fellow, APDI Fellow and Alexander von Humboldt Fellow. In 2012, Dr. Wu has been recruited to work in SIC, CAS, as One-Hundred Talent Program of Chinese Academy of Sciences. Then he was awarded Recruitment Program of Global Young Experts of China (One-Thousand Young Talent Program) and Shanghai Pujiang Talent Program. Prof. Wu’s research focuses on bioactive inorganic materials for bone tissue engineering and drug delivery application. Up to now, Prof. Wu has published more than 110 SCI peer-review journal papers, including Adv Funct Mater, Biomaterials (14 Papers), Adv Mater Interface, Small, J Control Release, J Mater Chem (15 Papers), Acta Biomater (22 Papers), ACS Appl Mater& Interface, Bone, Tissue Eng. etc. The papers have been cited more than 2300 times, H Index 31. Prof. Wu has been awarded 11 patents, in which three of them have been transferred to company.

***Comment:**** Regeneration of large-size bone defects represents a significant challenge clinically, which requires the used scaffolds with the stimulation of osteogenesis and angiogenesis for stem cells. It is known that microenvironments of bioscaffolds play an important role to stimulate tissue regeneration. How to design and prepare bioscaffolds with favorable microenvironments for tissue regeneration is one of interesting topics in the fields of biomaterials and tissue engineering. To establish a beneficial microenvironment of biomaterials, it is of great importance to combine both nutrient elements and biomimetic structure of scaffolds to stimulate osteogenesis and angiogenesis of stem cells. Besides the microenvironment of bioscaffolds, macrophage plays an important role to induce immunomodulatory microenvironment for further influencing osteogenesis. Therefore, it is suggested that scaffolds and osteoimmunomudulation-induced microenvironments should be considered for bone tissue engineering.*

## Xuenong Zou


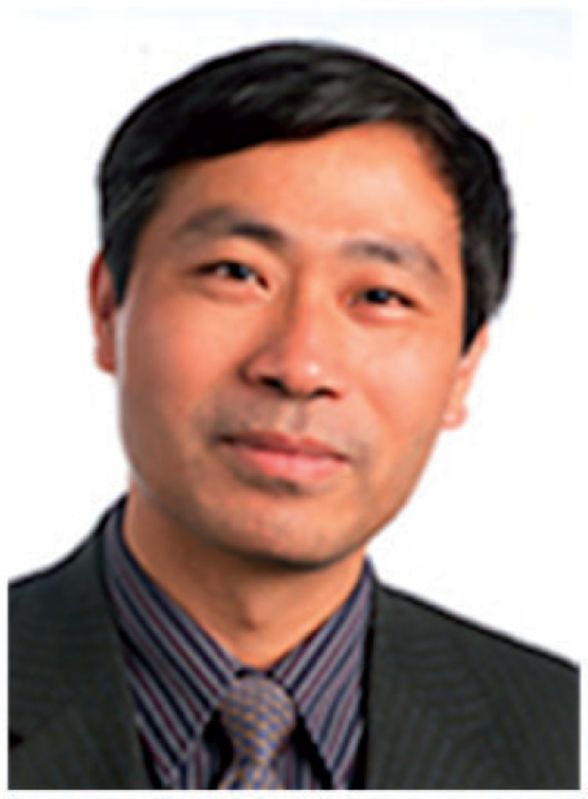


Dr. Zou is the director of the Orthopedic Research Institute and consultant surgeon of Spine Division at the First Affiliated Hospital of Sun Yat-sen University (SYSU). He graduated from Jiangxi Medical School, receiving the doctoral degree in Medical School of Aarhus University, Denmark. In 2008, Xuenong has been recruited as professor of the First Affiliated Hospital as One-Hundred Talent Program of SYSU. He has been focused on the interaction between biomaterials and host, the effect of ECM-biomaterials on bone formation, epigenetic mechanisms of osteogenetic progress induced by different biomaterials. His research has been supported by The State Key Program of National Natural Science Foundation of China, The National Key Basic Research Program (‘973’ Program) and The Key Program of NSFC-Guangdong Joint Fund, *et al.* Currently, he has published over 60 original research papers.

***Comment:**** There exist three sequential biological events after implantation of biomaterials—stress and homeostasis, immuno-inflammatory responses and bone formation or implantation failure. The clinical result of the implantation depends on the first two events, happening in the interaction between biomaterials and human body. Therefore, it’s very important to elucidate the roles and mechanisms of early biological events caused by implantation of biomaterials, as a guide for synthesizing and designing biomaterials.*

## Han Wu


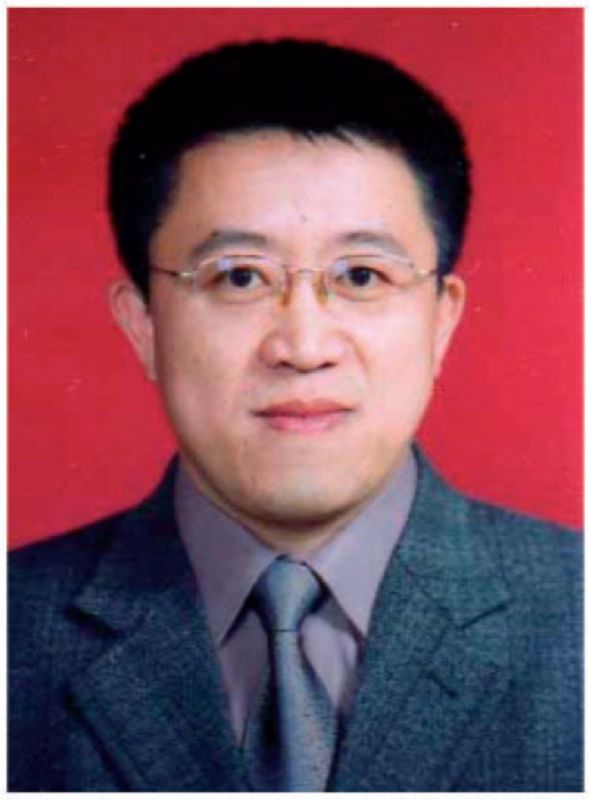


Professor of Department of Orthopedics in China-Japan Union Hospital, Jilin University, China. He graduated from the Norman Bethune Medical University in 1991. He got his Doctor degree from Jilin University in 2003. He had his visiting scholar experience in the University of New South Wales in Australia from 2008 to 2009. He has been engaged in orthopedic clinic for more than 20 years. His main research work is on spine surgery and bone tissue engineering and has published more than 60 papers. Now he is the member of the expert committee of Chinese national medical examination center, the member of the Chinese Medical Association Branch of minimally invasive Department of orthopedics and one of the China core members of Medtronic MMSG Association.

***Comment:*** *At present, there are about 3 million patients with defect bone or injured bone needed to be treated each year in China. More and more biological materials are demanded in orthopedic clinical work. To meet substantial clinical demand, with the high speed promotion of biomedical materials science and engineering, a high-tech biomedical materials and its products industry has already formed. But the present products of biomaterials can’t really biomimic the autologous grafts and the surgeons usually wondered that which was the best choice for a case when they face to a wide variety of products. Some of them will worry about the dissatisfied or even failed treatment and then refuse to accept the new products. One possible reason to affect the bone healing quality of a product might be the selection of operation indication or operation method but not the product itself. Thus, it is important for both researchers and surgeons that their frequent communication and cooperation will be conducive to the development of new biomaterials and the rapid development of orthopedic clinical technology.*
